# Behind the Leash: Burnout, Compassion Fatigue, and Occupational Strain in Dog Trainers

**DOI:** 10.3390/bs15060798

**Published:** 2025-06-10

**Authors:** Alexandra Malone

**Affiliations:** College of Psychology and Community Services, Walden University, Minneapolis, MN 55401, USA; alexandra.malone@waldenu.edu

**Keywords:** compassion fatigue, burnout, dog trainers, dog training, Professional Quality of Life Measure (ProQOL), compassion satisfaction, secondary traumatic stress, mental health interventions

## Abstract

The cases that dog trainers and behavior consultants face are often traumatic and emotionally challenging, especially under pressure from distressed clients. This study investigated whether more years of professional experience and higher levels of dog aggression contribute to burnout and whether the overall emotional toll of caring moderates these relationships. Eighty-six professionals completed the Professional Quality of Life Measure, which evaluates the positive aspects of caregiving and negative outcomes such as burnout and secondary traumatic stress. The analysis revealed that neither extended work experience nor increased dog aggression significantly predicted higher burnout levels, and the anticipated moderating effect of emotional strain was not observed. The levels of burnout and secondary traumatic stress in this sample were similar to those reported in other animal-care and human healthcare settings, while the satisfaction derived from caregiving remained relatively high. These results suggest that factors beyond years of experience and dog aggression—such as workplace support and individual coping mechanisms—may be more crucial in influencing burnout among these professionals. This study highlights the need for an expanded investigation of other possible influences, such as workplace support and personal coping mechanisms. Identifying specific challenges that dog trainers face and implementing strategies to offset burnout will create a healthier and more sustainable work environment and improve the quality of life for humans and their canine companions.

## 1. Introduction

Burnout and compassion fatigue are critical occupational hazards in emotionally demanding, high-stress caregiving and service roles. Burnout is a work-related syndrome characterized by chronic emotional exhaustion, depersonalization (cynicism or detachment), and a diminished sense of personal accomplishment (Maslach & Jackson, 1981; Schaufeli et al., 2009). Compassion fatigue, a concept related to secondary traumatic stress, refers to the emotional toll of repeatedly witnessing or empathizing with others’ suffering, leading to trauma-like symptoms and burnout-like exhaustion. Extensive research in healthcare, veterinary medicine, and social services links high job demands, emotionally charged work conditions, and prolonged exposure to stressors with elevated risks of burnout and compassion fatigue. Despite these well-documented findings, the dog training profession has received relatively little empirical attention, even as anecdotal reports suggest that dog trainers often experience significant emotional and occupational strain (Scotney et al., 2019; Thompson-Hughes, 2019). This study addresses that gap by focusing on burnout and compassion fatigue among dog trainers and behavior consultants.

Within the animal-care and welfare sector, professional dog trainers occupy a unique niche that blends behavior modification with extensive client interaction (Moses et al., 2018; Scotney et al., 2019). They are critical in rehabilitating severe canine behavior issues and fostering healthy human-dog relationships. However, their work conditions can be exceptionally challenging. Trainers frequently handle complex cases involving abused, anxious, or highly aggressive dogs, often while managing distressed owners’ expectations. They commonly work long hours under heavy workloads (Arluke, 2003; Hill et al., 2020; Reeve et al., 2005). This complex exposure to traumatized animals, emotional clients, and high job demands can heighten the risk of burnout and compassion fatigue, potentially compromising trainers’ well-being and the quality of services they provide. Similar stressors in other animal-care jobs have been linked to adverse mental health outcomes (Figley, 1995b; Scotney et al., 2015). However, systematic research on dog trainers’ occupational health remains scant (Scotney, 2017; Van Hooser et al., 2021).

In light of these challenges, two particular factors warrant investigation for their impact on burnout in dog trainers: years of professional experience and severity of dog aggression cases handled. The role of years of experience in burnout is debated in the broader caregiving literature. On one hand, seasoned professionals may develop better coping techniques and emotional boundaries over time, which could protect against burnout (Leiter & Maslach, 2004; Lloyd & Campion, 2017; Maslach et al., 2001). This perspective suggests that greater experience builds resilience, allowing long-time trainers to handle stress more effectively. On the other hand, prolonged exposure to stressful and emotionally taxing situations may accumulate and eventually overwhelm even experienced individuals, leading to chronic exhaustion and reduced job satisfaction (Figley & Roop, 2006; Fye et al., 2021). According to this view, each additional year in the field potentially adds to the “wear and tear” on practitioners, especially without adequate support. In the context of dog training, this dichotomy is especially pertinent. Experienced trainers might either benefit from hard-won resilience or suffer from the cumulative strain of managing difficult cases over the years. Clarifying whether more experience buffers against burnout or contributes to it can inform how to support trainers at different career stages.

The second factor of interest is the aggression level of dogs being trained. Working with highly aggressive or reactive dogs introduces both physical risks and emotional strain. Trainers handling severe aggression face intense pressure to ensure safety, meet client expectations, and navigate ethical dilemmas in behavior modification (Rank et al., 2009; Thompson-Hughes, 2019). In veterinary medicine and animal shelter work, exposure to aggressive or traumatized animals correlates with higher stress and burnout among staff (Scotney et al., 2019). Similar correlations might be expected for dog trainers regularly dealing with canine aggression. However, this specific relationship has not been empirically tested prior to the present study. It is conceivable that trainers who specialize in aggression cases experience greater emotional strain (due to safety concerns and high-stakes outcomes) and, thus, higher burnout. Conversely, it is also possible that those who choose to work with aggressive dogs have particular skills or support systems that help them cope. The lack of research on this topic represents a critical gap: understanding whether handling aggressive dogs is linked to trainer burnout is necessary for developing targeted interventions (for instance, specialized training or support for those working with high-risk cases).

A further layer of complexity is added by compassion fatigue, which may interact with the above factors. Compassion fatigue, often described as the cost of caring for others in emotional pain, can co-occur with or exacerbate burnout. Figley’s (1995a) model notes that professionals who continually empathize with clients (or, in this case, with animals and their owners) can undergo emotional depletion over time. In essence, the empathetic engagement that is central to effective dog training may also make trainers vulnerable to secondary trauma and exhaustion. High compassion fatigue can undermine a trainer’s ability to regulate emotions and employ coping strategies, thereby intensifying the impact of job stressors (Maslach & Leiter, 2008; Radey & Figley, 2007). Empirical studies in other helping professions have shown that elevated compassion fatigue is associated with greater emotional exhaustion and lower job satisfaction. For example, recent research on healthcare trainees and practitioners finds that those with higher compassion fatigue report more burnout symptoms (Chachula, 2022; Wardle & Mayorga, 2016). Given that dog trainers routinely work with traumatized animals and empathetically support distressed pet owners, it is plausible that compassion fatigue could amplify the effects of both experience and case severity on burnout. A trainer already prone to compassion fatigue may become especially exhausted as years in the field increase or as they handle more aggressive cases. Conversely, if a trainer has low compassion fatigue (perhaps due to strong self-care or support), that might buffer or protect them from burnout even when facing challenging conditions. The moderating role of compassion fatigue—whether it exacerbates or mitigates the impact of experience and aggressive caseload on burnout—is a central question of this research.

The interplay among these variables—professional experience, dog aggression severity, and compassion fatigue—presents a nuanced landscape that warrants comprehensive investigation. Previous studies in adjacent fields offer mixed expectations. Some findings in veterinary and human healthcare contexts suggest more experience might relate to lower stress or better outcomes (Beaumont et al., 2016; Tomasi et al., 2019), while other evidence points to burnout accumulating over time despite experience (Aronsson et al., 2017; Stevens & Al-Abbadey, 2023). There is consistent support that working with aggressive or trauma-exposed animals is stressful (Rank et al., 2009; Rohlf, 2018), but it is unknown if that directly translates to higher burnout in dog trainers, as this specific population has not been studied. Compassion fatigue adds complexity: it has the potential to either buffer or heighten burnout in conjunction with those factors. A more refined understanding of these dynamics is essential for developing effective support mechanisms and mental health interventions for professionals in this field. By identifying whether experience and case severity are true risk factors and how compassion fatigue influences outcomes, we can better tailor prevention and intervention efforts (such as training programs, counseling/coaching support, or organizational policy changes) to enhance dog trainers’ well-being.

Study Purpose and Hypotheses: The present study aims to examine how burnout among professional dog trainers and behavior consultants is influenced by (1) years of experience, (2) the aggression levels of dogs they work with, and (3) whether compassion fatigue moderates the strength of these relationships. In addressing these questions, this study targets a significant gap in the literature on occupational stress in animal-care professions. Based on the theoretical considerations and related research above, the following hypotheses were formulated:

**Hypothesis 1.**  *Burnout will vary with years of experience on the job among trainers and behavior consultants who offer obedience training and aggression training for dogs.*

**Hypothesis 2.**  *Burnout will vary among trainers and behavior consultants who offer obedience training and dog aggression training for dogs in relation to the aggression levels of target dogs.*

**Hypothesis 3.**  *Compassion fatigue does moderate the relationship between years of experience and burnout among trainers and behavior consultants who offer obedience training and aggression training for dogs.*

**Hypothesis 4.**  *Compassion fatigue does moderate the relationship between the aggression level of dogs to be trained and burnout among trainers and behavior consultants who offer obedience training and aggression training for dogs.*

**Hypothesis 5.**  *Compassion fatigue does moderate the relationship between two predictors: the aggression level of dogs being trained and years of experience of the trainers, and burnout among trainers and behavior consultants who provide obedience training and aggression training for dogs.*

Through testing these hypotheses, the study seeks to determine whether greater experience offers resilience or contributes to cumulative burnout when working with challenging dogs and to clarify the role of compassion fatigue in these processes. The findings are expected to provide nuanced insights into the occupational well-being of dog trainers. Importantly, by highlighting these relationships, this research can inform the development of targeted interventions to support dog training professionals. If certain factors (like lack of support or maladaptive coping) are found to be more influential than raw years of experience or case severity, then strategies can be implemented to improve those conditions, such as resilience-building training, peer support groups, coaching or mentoring programs, and organizational policies to reduce burnout risk. Ultimately, improving our understanding of burnout and compassion fatigue in dog trainers will help foster a healthier, more sustainable work environment for these professionals and enhance the quality of care for both their human clients and canine companions.

## 2. Materials and Methods

### 2.1. Research Design

This study employed a non-experimental, quantitative correlational design to investigate burnout and compassion fatigue in professional dog trainers and behavior consultants. The primary aim was to assess the relationships between two predictor variables (years of experience and dog aggression level) and the outcome variable (burnout) and to evaluate whether compassion fatigue serves as a moderating variable in these relationships. A correlational approach was appropriate because it allows the examination of natural relationships among variables without manipulating any conditions. The first two research questions (relating to experience and dog aggression) were addressed by using Pearson product-moment correlations, and the moderation hypotheses were tested by using multiple linear regression analyses with interaction terms. This combination of statistical techniques enabled us to determine the direct associations and the interactive effects of predictors and burnout.

### 2.2. Study Participants

The target population was defined as professional dog trainers and canine behavior consultants who utilize positive-reinforcement training methods. Participants self-identified as either “dog trainers” or “behavior consultants.” In the United States, these terms can denote distinct roles: “dog trainer” typically refers to individuals who provide skills training and basic obedience, while “behavior consultant” commonly describes professionals who address both training and more complex behavior modification, often requiring additional education or certification. The term “behaviorist” is generally reserved for individuals with advanced academic qualifications (e.g., MS, PhD) or recognized certification from bodies such as the Animal Behavior Society (ABS); however, no respondents in this study identified as “behaviorists.” For clarity and consistency, the term “training professionals” is used when referring to the full sample, and “trainer” or “behavior consultant” is used in accordance with participants’ self-reported titles and certifications where relevant. The focus was on positive-reinforcement training professionals to ensure a more homogeneous sample in terms of training philosophy and to capture those likely engaged in close emotional caregiving relationships with clients and animals. Participants were recruited in January 2024 using a convenience sampling strategy. Recruitment took place via online communities and professional networks: announcements were posted as digital flyers and invitation messages in several dog training groups, professional associations’ bulletin boards, and private social media pages for trainers. These invitations briefly described the study’s purpose and provided a link to an anonymous online survey. Participation was voluntary and open to any dog trainer/behavior consultant meeting the above criteria.

Before beginning the survey, individuals viewed an informed consent form that explained the study’s purpose, procedures, inclusion criteria, and potential risks/benefits and emphasized that participation was entirely voluntary. Participants had to agree to the informed consent before proceeding explicitly. They were informed that they could withdraw at any time without penalty, and no personally identifying information would be collected, ensuring anonymity and confidentiality. Given the potentially sensitive nature of burnout and compassion fatigue, the survey debriefing page provided resources for mental health support (such as links to counselor directories and helplines) in case the questionnaire raised any personal concerns. This precaution was taken to address any distress caused by reflecting on one’s burnout or secondary trauma. All procedures were approved by the Institutional Review Board of Walden University (Approval No. 01-08-24-0056135), ensuring compliance with ethical standards for research with human subjects.

A total of 119 individuals accessed the survey. Of these, 33 did not complete all required sections and were excluded from the analysis (partial responses were dropped). The final sample comprised N = 86 dog training professionals with complete data, yielding a completion rate of approximately 72%. An a priori power analysis indicated that a sample size of around 84 would be needed to detect a medium effect size (~0.3) with 80% power at α = 0.05 for the planned analyses (Faul et al., 2009). Thus, the obtained sample of 86 exceeded this threshold, suggesting the study was adequately powered. All participants self-identified as using primarily positive-reinforcement methods in their practice (100% of the sample), with none reporting the regular use of aversive training tools such as shock collars or dominance-based techniques. This aligns with the recruitment targeting. Participants first answered a set of demographic and background questions (e.g., age, gender, education, certifications, years of experience, types of services offered, and proportion of cases involving reactivity). In the survey, the term “reactivity” was used without a formal definition, allowing respondents to apply their own professional interpretation. In practice, “reactivity” can encompass a wide range of behaviors, from excitability and barking to overt aggression. For the purposes of analysis and reporting in this manuscript, we focused on cases involving aggression, defined as behaviors intended to cause harm or threat, and have used the term “aggression” throughout to ensure consistency and clarity. It should be noted that respondents may have interpreted “reactivity” differently based on their backgrounds and experiences. They then completed the standardized scales for burnout, compassion fatigue, and related constructs (described below). The online survey took approximately 15 to 20 min to complete. To avoid any potential coercion or undue influence, no compensation was offered for participation.

### 2.3. Ethical Considerations

This research adhered to strict ethical guidelines for human subjects. Institutional Review Board approval was obtained prior to data collection (Walden University IRB #01-08-24-0056135). Informed consent was obtained electronically from all participants, as described above, before they began the survey. Privacy and confidentiality were maintained by collecting data anonymously (no names or contact information were tied to survey responses). All data were stored securely in password-protected electronic formats accessible only to the researcher. Given the topic’s potentially sensitive nature, measures were implemented to minimize risk: Participants could skip any question they felt uncomfortable with, and the survey concluded with an informative debrief that included counseling and burnout support resources. Participants were also given the researcher’s contact information and the IRB’s contact details should they have any questions or concerns about the study. Throughout the study, ethical principles of voluntariness, beneficence, and confidentiality were upheld (Buchanan & Hvizdak, 2009; Evans & Mathur, 2005; Nayak & Narayan, 2019). No identifying information was collected, and results are reported only in aggregate form. There were no foreseeable physical risks to participants; the main ethical consideration was the possibility of emotional discomfort when reflecting on one’s work stress, which was mitigated through the above support measures. Ultimately, the knowledge gained from this research is intended to benefit the profession by informing strategies to improve trainers’ well-being, justifying the minimal risks involved.

### 2.4. Measurement Tools

#### 2.4.1. Demographic and Professional Background

A custom questionnaire was used to gather demographic information (e.g., age, gender identity, education level) and professional background data. Key items included years of experience as a dog trainer/behaviorist consultant (self-reported total years practicing), primary training methods used (with a checklist of methods, allowing multiple selections to verify the use of positive reinforcement), and exposure to dog aggression cases. To quantify exposure to canine aggression, respondents were asked what proportion of their current caseload involved reactivity cases and were prompted to indicate the typical severity of the cases they handle. This was operationalized using an adaptation of Dunbar’s Dog Bite Scale (American Professional Dog Trainers Association [APDT], 2017) as described below. Additional questions covered professional certifications (e.g., CPDT-KA, KPA-CTP, IAABC credentials), and the types of services offered (basic obedience, separation anxiety, rehabilitation, etc.). These background variables provided context for the sample and were examined in descriptive analyses to characterize the trainers’ professional profiles.

#### 2.4.2. Professional Quality of Life Scale (ProQOL Version 5)

Burnout, compassion fatigue, and compassion satisfaction were measured with the Professional Quality of Life Scale, Version 5 (ProQOL5). The ProQOL is a widely used self-report instrument that assesses both the positive and negative effects of helping others (Stamm, 2010). It consists of 30 items rated on a 5-point Likert scale from 1 (“Never”) to 5 (“Very often”), covering three subscales: Compassion Satisfaction (CS), Burnout (BO), and Secondary Traumatic Stress (STS). Compassion satisfaction reflects the positive feelings derived from one’s work helping others (e.g., enjoyment and sense of accomplishment in the role). Burnout reflects work-related hopelessness and feelings of inefficacy (e.g., feeling exhausted or discouraged about one’s work). Secondary traumatic stress (often used interchangeably with compassion fatigue in this context) reflects work-related exposure to extremely stressful or traumatic events (e.g., feeling overwhelmed by others’ traumatic experiences). Each subscale comprises 10 items. Example items include “I feel worn out because of my work as a helper” (Burnout), “I feel I am positively influencing other people’s lives through my work” (Compassion Satisfaction), and “I feel as though I am experiencing the trauma of someone I have [helped]” (Secondary Traumatic Stress). Subscale scores are obtained by summing relevant items (after reverse-scoring specified items); higher scores indicate greater levels of the construct. For interpretation, Stamm (2010) provides guideline cutoffs: scores ≤ 22 indicate low levels, 23–41 indicate moderate, and ≥42 indicate high levels of Burnout, Compassion Satisfaction, or STS.

The ProQOL has well-established psychometric properties. It has been validated in diverse helper populations, including healthcare providers, social workers, counselors, veterinarians, and animal welfare workers (Heritage et al., 2018; Sprang et al., 2011; Stamm, 2010). In this study, the ProQOL demonstrated good internal consistency: Cronbach’s alpha was α = 0.75 for the Burnout subscale, α = 0.81 for Secondary Traumatic Stress, and α = 0.88 for Compassion Satisfaction, indicating acceptable to high reliability. These values are consistent with prior research (typically α ~0.7–0.8 for burnout and secondary traumatic stress and > 0.8 for compassion satisfaction). The ProQOL’s burnout scale served as the primary outcome measure.

In this study, compassion fatigue (CF) was operationalized using the Secondary Traumatic Stress (STS) subscale of the Professional Quality of Life Scale (ProQOL). The ProQOL, as described by its developer (Stamm, 2010), measures three related constructs: compassion satisfaction, burnout, and secondary traumatic stress. While the scale distinguishes these subscales, many empirical studies in helping professions have used the STS subscale as a proxy for compassion fatigue, as reviewed by Bride et al. (2007) and exemplified by studies in nursing and emergency care (Beck, 2011; Dominguez-Gomez & Rutledge, 2009). However, it is important to note the evolving conceptual distinction in the literature: Whereas Figley (1995a) often used the terms STS and CF interchangeably, Stamm’s (2010) more recent work conceptualizes compassion fatigue as a broader construct, encompassing both STS (an individual’s personal response to secondary trauma) and elements of work-related burnout, along with organizational and systemic factors. Thus, the use of the STS subscale in this study primarily captures the individual, trauma-related aspect of compassion fatigue, rather than its full organizational and contextual dimensions. This partial operationalization should be considered when interpreting the findings, and future research may benefit from employing additional measures or frameworks to address the broader, organizational components of compassion fatigue (Heritage et al., 2018; Hinderer et al., 2014; Sinclair et al., 2017). Compassion satisfaction scores were examined for descriptive context (to see how positive aspects of work might counterbalance the negatives) but were not focal to the hypotheses.

#### 2.4.3. Dunbar’s Dog Bite Scale

To assess the aggression level of dogs each trainer typically works with, the Dunbar’s Dog Bite Scale (American Professional Dog Trainers Association [APDT], 2017) was adapted for this study. Dunbar’s scale categorizes dog bites/attacks into six levels of severity: Level 1 involves aggressive behavior with no skin contact by teeth (threatening gestures only), Level 2 is teeth making contact with skin but no puncture, Level 3 includes one to four shallow punctures, Level 4 involves one to four deep punctures (at least one puncture deeper than half the length of the dog’s canine tooth), Level 5 involves multiple bites with deep punctures (a sustained attack), and Level 6 is a fatal attack. For the survey, trainers indicated the highest bite level of cases they *regularly* handle and the approximate proportion of their cases that fall into each level category. Many respondents reported a range of cases; thus, for analysis, an Aggression Exposure Index was created by combining frequency and severity information. Specifically, the bite-level exposure was recoded into three categories for simplification: 0 = No Aggression Cases (trainers who reported handling no aggressive dogs in their work), 1 = Mainly Low-Level Aggression (trainers whose caseload consists of at least 50% Level 1 cases and minimal higher-level cases), and 2 = Regular High-Level Aggression (trainers who handle a substantial proportion—50% or more—of Level 2 or higher cases, indicating routine work with dogs that have bitten and/or caused injury). This categorical variable was used in analyses to compare burnout across different aggression exposure levels. The rationale was to distinguish those who rarely/never deal with aggression, those who deal mostly with mild aggression, and those who frequently deal with moderate to severe aggression, as these groups might differ in stress outcomes. This approach mirrors methods in shelter worker studies that classify employees by frequency of euthanasia or aggression cases handled (e.g., high-exposure vs. low-exposure groups) to examine burnout differences (Rohlf, 2018; Scotney et al., 2019). While the study’s measure was self-reported and coarse, it provides a novel quantitative estimate of the aggression-related risk in each trainer’s workload.

### 2.5. Statistical Analysis

The study employed descriptive and inferential statistical analyses to examine the relationships between burnout, years of experience, and the aggression levels of dogs trained while assessing compassion fatigue as a moderating variable. All analyses were conducted using IBM SPSS Statistics for Windows Version 29.0 (IBM Corp, 2023).

Before conducting the main statistical tests, several assumptions were assessed. Normality was tested using the Kolmogorov–Smirnov (K-S) test, multicollinearity was examined through Variance Inflation Factors (VIFs) and Tolerance Statistics, and homoscedasticity was assessed using scatterplots of standardized residuals. These checks ensured that the data met the assumptions necessary for correlation and regression analyses (Micheal & Abiodun, 2014).

Descriptive statistics, including means, standard deviations, and frequency distributions, were calculated to summarize the characteristics of the study sample. This included participant demographics, years of experience, exposure to dog aggression levels, and burnout and compassion fatigue scores. Pearson correlation coefficients were computed to assess the strength and direction of the relationships between years of experience and burnout, dog aggression levels (bite scale) and burnout, and compassion fatigue and burnout. These correlations helped determine whether further regression analysis was warranted. Hierarchical multiple regression analyses were conducted to evaluate the predictors of burnout and the moderating effect of compassion fatigue. The independent variables included years of experience (YE), dog aggression level (bite level, BL), and compassion fatigue (CF) as moderating variables.

Three regression models were tested:

(1) Burnout as the dependent variable, with years of experience as the predictor
(2) Burnout as the dependent variable, with dog aggression level as the predictor
(3) Burnout as the dependent variable, with interaction terms testing the moderating effect of compassion fatigue

The regression models provided insights into which factors were significant predictors of burnout and whether compassion fatigue moderated these relationships. Applying these statistical techniques, the study aimed to comprehensively analyze burnout among dog trainers and behavior consultants, identifying key risk factors and potential intervention points.

## 3. Results

### 3.1. Demographic Characteristics of Participants

A total of 86 dog training professionals provided complete data. Key demographics of the sample are as follows: the majority of respondents identified as female (81.4%, n = 70), with 7.0% (n = 6) identifying as male, 3.5% (n = 3) as transgender, 5.8% (n = 5) as non-binary, and the remainder either self-described their gender or preferred not to answer. The ages of participants ranged from the early 20s to late 60s (exact age was optional; 70.9% reported an age, with a median in the mid-30s). In terms of education, over half of the sample (approximately 51%) held a bachelor’s degree or higher (27.9% with a master’s, 1.2% with a doctorate), 9.3% had an associate degree, 5.8% had some college education, and 4.7% had other training (such as specialized certifications). Notably, almost all participants (88%) possessed at least one professional certification in dog training or behavior (e.g., CPDT-KA), and about one-third held two or more certifications, reflecting a highly trained cohort.

In terms of professional practice, all respondents were positive-reinforcement trainers or behavior consultants by selection criteria. None reported using aversive methods like shock or prong collars as a primary technique, and many incorporated multiple humane training methodologies. For example, 95% indicated using reward-based positive reinforcement, 51% used clicker training, and about 20% mentioned other non-aversive methods (note: these percentages sum to over 100% because multiple responses were allowed). Many trainers offered a mix of services: 73.3% provided both obedience training and behavior modification for different issues, whereas a smaller fraction specialized exclusively in one area (about 11.6% focused only on basic obedience). On average, participants reported 12.98 years of experience in dog training (SD = 9.80, range 1 to 40 years), indicating a wide range from newcomers to very seasoned professionals. The median experience was around 10 years, with 21% of the sample being relatively early-career (≤5 years of experience) and a substantial portion (approximately 30%) having over 15 years in practice. This distribution allowed us to examine effects across a spectrum of experience levels (see Table 1).

Regarding exposure to canine aggression, by the earlier defined categories, 19.8% of the trainers (n = 17) fell into category 0 (they reported that they do not deal with aggression cases in their work—likely focusing on obedience and general training only). Around 52.3% (n = 45) were in category 1, meaning they handle some aggressive dogs but primarily of lower severity (e.g., most cases involve Level 1 bites or threats without injury). The remaining 27.9% (n = 24) were categorized as high aggression exposure (category 2), regularly working with moderate to severe aggression cases (Level 2–5 bites). No participant reported handling Level 6 (fatal attack) cases as a routine part of their work, which is expected since those cases would typically involve legal/animal control interventions beyond training. These figures highlight that about one-quarter of the sample frequently works with dogs capable of causing significant injury, while the rest have little to moderate exposure to such high-stakes cases.

A cross-tabulation analysis was conducted to examine the association between advanced certification (CBCC-KA) and aggression case exposure (see Table 2). None of the CBCC-KA-certified professionals reported having exclusively non-aggression cases, while 25.8% of trainers without a CBCC-KA credential did. Among CBCC-KA-certified professionals, 75% reported mainly low-level aggression cases, and 25% reported regular high-level aggression cases. Among trainers without a CBCC-KA credential, 53% reported mainly low-level aggression cases, and 21.2% reported regular high-level aggression cases. The association between behavioral certification status and aggression case exposure was statistically significant (Pearson Chi-square = 6.53, df = 2, *p* = 0.038; Fisher-Freeman-Halton exact test, *p* = 0.022). These findings suggest that CBCC-KA-certified professionals are more likely to handle at least some aggression cases compared to those without this credential.

### 3.2. ProQOL Scores and Descriptive Statistics

Table 3 summarizes the ProQOL subscale scores (Burnout, Secondary Traumatic Stress, and Compassion Satisfaction) in the sample, along with internal consistency (Cronbach’s α). Overall, the trainers reported moderate levels of burnout and secondary traumatic stress and moderate-to-high levels of compassion satisfaction. Burnout scores ranged from 15 to 35 (out of a possible 50), with a mean of 25.41 (SD = 4.98). According to the ProQOL categorization, 30.2% of participants’ burnout scores fell in the “low” range (22 or below), 69.8% were “moderate” (23–41), and notably, none of the participants scored in the high burnout range (42 or above). The highest observed burnout score was 35, comfortably below the high cutoff, suggesting that severe burnout was not present in this sample despite many reporting notable stress.

For Secondary Traumatic Stress, scores ranged from 11 to 41 (out of 50), with a mean of 26.66 (SD = 6.51). Using the same cutoffs, about 24.4% of respondents were in the low secondary traumatic stress range, 75.6% in moderate, and none scored in the high secondary traumatic stress range (the highest secondary traumatic stress score observed was 41, right at the upper bound of moderate). This indicates that while secondary trauma symptoms were present for many (three-quarters had at least moderate levels), extremely high compassion fatigue was not common in this group. Participants did experience some intrusive thoughts or emotional distress related to their work (as captured by secondary traumatic stress items), but few reached the levels often seen in, for example, frontline emergency workers or trauma therapists.

Compassion Satisfaction scores were relatively high: they ranged from 29 to 49 (out of 50) with an average of 40.12 (SD = 5.22). Interpreted via Stamm’s criteria, 65.2% of trainers scored in the moderate range for compassion satisfaction, 34.8% scored high, and none scored in the low range. Most participants derived a great deal of satisfaction and meaning from their work with dogs and clients. This is an encouraging finding, as high compassion satisfaction can serve as a protective factor against burnout. Many trainers agreed with positive statements like “I get satisfaction from being able to help [dogs/clients]” and “I feel invigorated after working with those I help,” reflecting a strong sense of professional reward. These positive feelings coexisting with moderate burnout suggest that even though the work is taxing, it is also deeply fulfilling for most respondents. Internal consistencies for the subscales in the sample were α = 0.888 for Burnout, 0.839 for secondary traumatic stress, and 0.731 for compassion satisfaction (noted in Table 2), which are acceptable and comparable to established norms. The lower alpha for compassion satisfaction (0.73) is typical as that scale often has slightly more varied item content. This pattern is consistent with prior psychometric evaluations of the ProQOL, which have frequently reported somewhat lower internal consistency for the Compassion Satisfaction subscale compared to burnout and secondary traumatic stress. For example, Stamm (2010) found alphas ranging from 0.84 to 0.90 for burnout and secondary traumatic stress, but typically around 0.88 or lower for Compassion Satisfaction; other studies report compassion satisfaction alphas in the 0.70s or low 0.80s (Bride et al., 2007; Heritage et al., 2018). This has been attributed to broader item content or greater diversity in positive work-related experiences among respondents.

In sum, the ProQOL results depict a profile where dog trainers experience a moderate amount of work-related stress and secondary trauma but also maintain a high level of professional satisfaction and have not generally lapsed into severe burnout. This balance will be further examined in relation to the key predictors. As shown in Table 2, mean burnout and secondary traumatic stress scores fell well within the moderate range, and the mean compassion satisfaction score was relatively high (close to the high threshold). These results will be revisited in the Discussion, especially compared to findings from other caregiving professions.

### 3.3. Correlation Analysis

First, simple bivariate correlations were examined to test the primary relationships of interest (Hypotheses 1 and 2). Years of experience did not show a significant correlation with burnout, *r*(86) = −0.080, *p* = 0.463. Similarly, another Pearson correlation coefficient was calculated to evaluate the relationship between burnout and aggression levels in dogs, which also showed that the correlation was not significant, *r*(86) = 0.055, *p* = 0.614. A summary of these results is presented in Table 4.

### 3.4. Multiple Regression Analysis

A multiple regression analysis examined the moderating effects within the proposed model, with burnout as the dependent variable. Predictor variables included compassion fatigue, years of experience, and their interaction term. The regression model was statistically significant, explaining a substantial portion of the variance in burnout (*R*2 = 0.924, *F*(3, 82) = 160.226, *p* < 0.001; see Table 5 for detailed regression analysis results).

The direct effect of compassion fatigue on burnout was significant (*B* = 0.005, *p* < 0.001, *sr*^2^ = 0.2313), positive, and accounted for approximately 23% of the variability in burnout. However, the direct effect of years of experience on burnout was not significant (*B* = 0.049, *p* = 0.450, *sr*^2^ = 0.0021). After controlling for the significant main effects of years of experience and compassion fatigue, the interaction between compassion fatigue and years of experience was not significant (*B* = 2.910 × 10^−5^, *p* = 0.278, *sr*^2^ = 0.0021). These findings indicate that compassion fatigue does not significantly moderate the relationship between years of experience and burnout among dog trainers.

A similar multiple regression analysis was performed to examine the moderating effects within the proposed model, with burnout as the dependent variable. Predictor variables included compassion fatigue, bite level, and their interaction term. The regression model achieved statistical significance, accounting for a substantial portion of the variance in burnout (*R*^2^ = 0.858, F(3, 82) = 165.809, *p* < 0.001; refer to Table 6 for detailed regression analysis results).

The direct effect of compassion fatigue on burnout was significant (*B* = 0.005, *p* < 0.001, *sr*^2^ = 0.2948), positive, and accounted for approximately 29% of the variability in burnout. Conversely, the direct effect of BL on burnout was not significant (*B* = −0.716, *p* = 0.445, *sr*^2^ = 0.0010). After controlling for the significant main effects of bite level and compassion fatigue, the interaction between compassion fatigue and bite level was not significant (*B* = 2.910 × 10^−5^, *p* = 0.278, *sr*^2^ = 0.0021). These results suggest that compassion fatigue does not play a significant moderating role in the relationship between bite level and burnout in dog trainers.

An additional multiple regression analysis was conducted to explore the combined moderating effects within the proposed model, with burnout as the dependent variable. Predictor variables included compassion fatigue, years of experience, bite level, and interaction term. The regression model achieved statistical significance, explaining a large proportion of the variance in burnout (*R*^2^ = 0.862, *F*(4, 81) = 126.927, *p* < 0.001; see Table 7 for detailed regression analysis results).

The direct effect of compassion fatigue on burnout was significant (*B* = 0.005, *p* < 0.001, *sr*^2^ = 0.5595), positive, and accounted for approximately 56% of the variability in burnout. The direct effect of bite level on burnout was also significant (*B* = −1.044, *p* = 0.020, *sr*^2^ = 0.0096), negative, and accounted for approximately 0.96% of the variability in burnout. The direct effect of years of experience on burnout was not significant (*B* = −0.010, *p* = 0.762, *sr*^2^ = 0.0001).

After controlling for the significant main effects of bite level, years of experience, and compassion fatigue, the interaction among compassion fatigue, bite level, and years of experience was not significant (*B* = 1.053 × 10^−5^, *p* = 0.242, *sr*^2^ = 0.0024). These findings suggest that compassion fatigue does not significantly moderate the relationship between bite level, years of experience, and burnout among dog trainers. The results of these analyses are summarized in Table 8.

## 4. Discussion

The present study investigated burnout and compassion fatigue in dog trainers and behavior consultants, focusing on how these outcomes relate to trainers’ years of experience and the aggression levels of the dogs they work with. Also examined was whether compassion fatigue (secondary traumatic stress) moderates the impact of experience and aggressive caseload on burnout. Five specific hypotheses were suggested, and the results can be summarized as follows: neither years of experience nor dog aggression levels showed a significant association with burnout (H1 and H2 were not supported), and compassion fatigue did not moderate the relationship of burnout with experience (H3 not supported) nor with aggression level (H4 not supported). Additionally, no significant three-way interaction was found among experience, aggression, and compassion fatigue on burnout (H5 not supported). The quantitative findings did not confirm any of the expected direct or moderating effects—aside from the strong link between compassion fatigue and burnout, which, while not a hypothesized interaction, underscores the close relationship between those two constructs. Despite the lack of support for the specific hypotheses, these findings are informative and somewhat surprising. Below, each key result is discussed in the context of existing literature, with possible explanations and practical implications. The sample’s profile is also compared to other related professions, and strategies to mitigate burnout and compassion fatigue in dog trainers are discussed.

### 4.1. Relationship Between Burnout and Years of Experience

It was hypothesized that burnout would vary with the number of years a trainer has been in the profession, expecting burnout to increase as experience accumulates (based on cumulative exposure to stress). However, the results did not show a significant relationship between years of experience and burnout. The correlation was near zero, and even when considering a potential interaction with compassion fatigue, years of experience remained a non-significant factor. While there was a slight trend in the raw data for burnout scores to be higher among more experienced trainers, this trend was weak and not statistically reliable. Thus, Hypothesis 1 was not supported by the evidence.

This result aligns with some prior observations in people-helping fields that burnout does not simply increase linearly over time (Bartram & Baldwin, 2010; Van Mol et al., 2015). Burnout is a multifaceted syndrome, and one’s career length might not capture the quality of experiences or coping mechanisms that develop over time. One possible explanation is the phenomenon of self-selection or survivor bias in the field (Chowdhury et al., 2017). Those individuals who were highly susceptible to burnout may have left the profession earlier in their careers, whereas those who remain long-term might inherently have better resilience or have developed effective coping strategies (Levitt & Gezinski, 2020; Robino, 2019). The sample with very experienced trainers could be biased toward more burnout-resistant people. This “healthy worker effect” is recognized in many professions: the people who stay for decades often have found ways to manage the stress, whereas those who burned out may have changed careers or scaled back (Chowdhury et al., 2017). This could flatten the observed relationship between experience and burnout in a cross-sectional snapshot.

Another consideration is that experience can confer both advantages and disadvantages. As suggested in the introduction, experienced trainers might have greater mastery of skills, confidence, and established client bases, potentially reducing some work stressors (Leiter & Maslach, 2004; Maslach et al., 2001). They may have learned how to distance themselves when emotionally necessary and how to employ efficient training techniques, thereby avoiding some frustration and fatigue. On the other hand, veteran trainers may also have taken on more responsibilities, tougher cases, or leadership roles that introduce new stressors (like running a business, supervising others, or managing a reputation). The net effect could be zero. The data hint that any increase in burnout over the years might be offset by those who manage to cope or those who leave if they cannot. This complexity suggests that years of experience alone is an insufficient predictor of burnout—individual differences (like personality, coping style, and support systems) and organizational factors likely play a moderating role (Hinderer et al., 2014; Sinclair et al., 2017). Organizational factors can be equally or even more influential than personal characteristics and include high caseloads, inadequate staffing, limited access to supervision or professional support, low perceived control, unclear job expectations, workplace incivility, and organizational policies that may fail to recognize or mitigate emotional demands (Bride, 2007; Hunsaker et al., 2015). Conversely, a positive organizational culture, opportunities for professional development, peer support, and formal debriefing processes can serve as protective factors against compassion fatigue in both healthcare and animal welfare settings. Given that the present study did not measure such organizational variables, future research should consider a more comprehensive assessment to identify both personal and systemic targets for intervention.

It is also worth noting that the sample did include a fair number of relatively inexperienced trainers (about 21% had ≤5 years of experience). It is possible that burnout levels start moderate in the early years (due to learning challenges, lower confidence, etc.), then might dip or stabilize in mid-career as people adjust, and perhaps rise again later due to other factors (like physical tiredness or boredom). A simple linear correlation would not capture such a non-linear pattern. Some studies in other fields have found mixed results—for example, newcomers can be overwhelmed and burn out quickly, whereas mid-career professionals might cope better, and then late-career professionals might experience stagnation or fatigue again (Shanafelt et al., 2022). Although the data did not explicitly test a curvilinear trend, a visual inspection did not suggest a strong U-shape, but future research could examine this possibility with larger samples.

The lack of a clear experience–burnout link underscores that longevity in the dog training field does not guarantee burnout or immunity from it. Burnout appears to be more idiosyncratic, likely influenced by personal and situational factors rather than simply time on the job. This finding highlights the importance of supporting trainers throughout their careers—interventions should not just target newcomers (assuming they are most at risk) or veterans (assuming burnout accumulates) but rather be available to anyone showing signs of strain. It also suggests that further research should explore what differentiates those who thrive long-term from those who struggle beyond just years in practice.

### 4.2. Relationship Between Burnout and Dog Aggression Levels

The expectation was that trainers who work with higher levels of dog aggression would experience higher burnout, given the added pressures and risks of those cases. Contrary to this expectation, the results did not show a significant relationship between aggression exposure and burnout. Trainers who frequently dealt with aggressive dogs did not report higher burnout on average than those who rarely or never did. Hypothesis 2 was thus not supported. This finding is somewhat surprising, as literature from animal shelter workers and veterinarians often points to certain work content (like euthanasia or severe injury cases) being associated with stress and emotional exhaustion (Rohlf, 2018; Scotney et al., 2019). Why might dog aggression not play a major role in trainer burnout, according to the data?

One interpretation is that trainers who handle a high workload of aggression cases may have developed specialized skills and coping mechanisms that buffer them against burnout. Dealing with aggressive dogs requires expertise in behavior management and safety protocols. Those who choose to work extensively with such dogs might have higher self-efficacy and confidence in their abilities, which can mitigate stress. Research in related fields suggests that feeling effective and competent in handling difficult tasks can reduce the impact of job stressors on burnout (Stevens & Al-Abbadey, 2023; Thompson-Hughes, 2019). In this context, a trainer who successfully rehabilitates aggressive dogs or prevents bites might derive a sense of accomplishment that counterbalances the stress. This ties into compassion satisfaction—which was relatively high in the sample—potentially offsetting burnout even in difficult aggression cases.

Another factor could be social and professional support. Trainers dealing with aggression cases may seek out mentorship, continuing education, or peer networks (e.g., forums and support groups of trainers who handle aggression) to help them navigate these challenges (Reeve et al., 2005). If they have colleagues to consult or share experiences with, this collegial support can alleviate feelings of isolation and stress. Conversely, trainers who work in isolation with difficult cases could be more at risk. Notably, many trainers in the sample indicated membership in professional organizations and certifications, which often come with communities of practice. Such a community could soften the impact of burnout. Indeed, studies show that professionals in high-stress jobs are more resilient when they have strong peer support systems and a sense of not facing challenges alone (Figley & Roop, 2006; Mastenbroek et al., 2014). The findings may indirectly reflect that those high-exposure trainers have tapped into those support resources.

It is also possible that personal passion plays a role. Trainers who take on aggressive cases might do so out of a strong passion for helping “the tough ones,” which could imbue their work with greater meaning. Studies in animal-care professionals found that viewing one’s work as fulfilling a personal mission (a “lifelong dream”) was protective against compassion fatigue (Reeve et al., 2005; Rohlf & Bennett, 2005). It may be that trainers specializing in aggression see it as a calling, and this sense of purpose shields them from burnout to some extent. In contrast, trainers who do not handle aggression might face other stressors (like client compliance issues with training or financial stress from limiting their services) that can also cause burnout. In other words, different stressors might affect trainers who do aggression vs. those who do not, but the overall burnout outcome could end up similar in magnitude for different reasons.

Interestingly, when controlling for compassion fatigue and experience in the full regression, aggression exposure showed a very small negative effect on burnout (those with more aggression cases had slightly lower burnout). While there is little weight on that finding (it was very small), it hints that perhaps those trainers have certain protective characteristics (as discussed). This might also reflect that some trainers not dealing with aggression might nonetheless experience burnout from things like business pressures or monotony—factors not measured here.

It is important to note that organizational context could moderate the effect of case type on occupational well-being. For example, a trainer employed at a large facility or shelter and handling aggressive dogs might have access to more formal support systems (such as team debriefings, peer collaboration, and established safety protocols) or, conversely, might face additional pressures due to higher caseloads and management demands. In the present sample, employment status (self-employed versus employed by an organization) was not explicitly assessed. However, based on years of experience and industry norms, it is likely that many respondents were independent trainers or business owners, where organizational structures and available supports may differ from those in larger facilities or shelters. Professionals in high-stress environments without adequate organizational support are known to be at greater risk for burnout and compassion fatigue (Hunsaker et al., 2015; Scotney et al., 2019). If some trainers handling high-aggression cases lack access to organizational support, they may be more vulnerable to burnout; however, the present study cannot directly address this distinction due to the lack of explicit data on employment context. Future research should specifically assess organizational context and employment status to clarify how these factors interact with case type and affect the risk for compassion fatigue and burnout.

The lack of a direct link between aggression cases and burnout suggests that simply the content of one’s caseload (aggressive vs. non-aggressive dogs) is not the sole determinant of well-being. The way those cases are managed, the trainer’s sense of competence, the support available, and perhaps personal inclination all likely influence how stressful those cases become. The findings encourage a more nuanced view: rather than assuming certain case types will automatically cause burnout, we should identify what resources or traits enable trainers to handle those cases without burning out. Those could include specialized training in handling aggression, opportunities to debrief difficult cases with peers or mentors, and ensuring that trainers have appropriate safety and referral protocols so they do not feel unsupported when dealing with dangerous situations.

### 4.3. Moderating the Role of Compassion Fatigue

It was explored whether compassion fatigue (secondary traumatic stress) would amplify the relationships between the above factors (experience, aggression) and burnout. Compassion fatigue itself was strongly correlated with burnout, reflecting that trainers who internalize the emotional suffering of clients or animals tend to feel more exhausted and cynical—a pattern well-documented in caregiving fields (Bride, 2007; Cieslak et al., 2014). However, the moderation analyses found no significant interaction effects: high compassion fatigue increased burnout levels, but it did so relatively uniformly across all levels of experience and aggression exposure. Hypotheses 3 and 4, predicting moderation, were thus not supported. This indicates that the impact of compassion fatigue on burnout in the sample is more of an additive effect rather than a conditional one. One interpretation is that compassion fatigue is a pervasive risk factor that can lead to burnout irrespective of other job characteristics. If a trainer is experiencing secondary traumatic stress (symptoms like being haunted by traumatic images of cases, feeling emotionally numb or overly empathetic distress), that alone can drain their emotional energy and lead to burnout (Maslach & Leiter, 2008). The data suggest this is happening across the board: those with high compassion fatigue had high burnout.

The lack of moderation might also imply that factors other than compassion fatigue are more critical in determining the relationships between experience, aggression, and burnout. The nonsignificant interactions could mean that the effects of years of experience and aggression (which were minimal, to begin with) are not significantly altered by compassion fatigue levels. It is possible that other variables—such as work–life balance, social support, or job control—play a moderating role between experience and burnout, as suggested in some literature (Fye et al., 2021; Stevens & Al-Abbadey, 2023). For instance, an experienced trainer with poor work–life balance might burn out more, whereas an equally experienced trainer with good balance might not—and that difference might have little to do with compassion fatigue per se. Similarly, the effect of handling aggression could be buffered by having a supportive supervisor or colleague (unrelated to one’s personal compassion fatigue level).

It is also worth considering measurement nuances: the secondary traumatic stress scale of ProQOL was used as the indicator of compassion fatigue. While often accepted, some argue that compassion fatigue encompasses both secondary trauma and burnout symptoms (Figley, 1995b), making it conceptually and empirically very close to burnout itself. In fact, some overlap in item content exists (though ProQOL attempts to separate them). The high correlation between compassion fatigue and burnout could partly be due to item overlap or shared variance (Bride et al., 2007). In moderation terms, when an interaction is attempted between two highly correlated predictors, it can be statistically challenging to detect unless the moderation effect is large. The non-significant interaction might thus reflect that most variance was already taken up by the main effects. Future research could consider alternative measures or approaches (e.g., splitting the sample by compassion fatigue level to see if any pattern emerges, albeit that reduces power).

Nonetheless, the practical takeaway is that compassion fatigue is a serious concern. Trainers experiencing compassion fatigue are at high risk of burnout, regardless of their tenure or clientele. This underscores the importance of addressing compassion fatigue directly. Strategies to help trainers manage secondary traumatic stress (such as counseling, trauma-informed supervision, or training in boundaries and detachment) could be universally beneficial in this field rather than tailoring those strategies only to certain subsets of trainers. In the sample, compassion fatigue’s effect did not discriminate—suggesting that any trainer, new or seasoned, specialist or generalist, can succumb to it if not properly supported. This finding aligns with the notion that compassion fatigue is an “occupational hazard” of caring professions that needs to be consistently monitored and mitigated across the workforce (Figley & Roop, 2006).

The absence of the expected moderation effects indicates that the relationships among the variables are more complex than in the initial model. It might be that other moderating factors exist. For example, personal resilience traits (like hardiness or optimism) or external support (like mentorship availability) could moderate how experience translates to burnout—these were not measured in the study but are highlighted in the literature (Fye et al., 2021; Stevens & Al-Abbadey, 2023). It is also possible that the effect of compassion fatigue on burnout might be moderated by variables that were not tested—say, trainers’ use of self-care practices or their caseload volume. While compassion fatigue was not found to act as a moderator, it remains possible that burnout is a multidimensional outcome that needs a multifactorial model to be fully explained.

### 4.4. Comparison with Other Animal-Related Professions and Healthcare Providers

**Figure 1:**
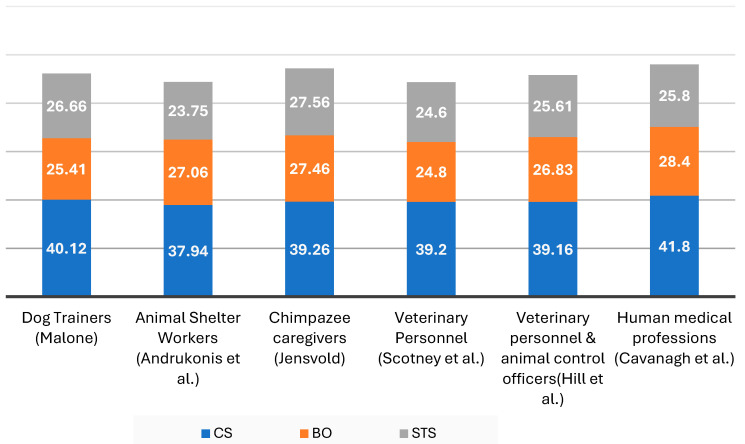
ProQOL scales comparison for mean scores between dog trainers, animal shelter workers, veterinary personnel, chimpanzee caregivers, and human medical professionals (Malone, 2024; Andrukonis et al., 2020; Jensvold, 2022; Scotney et al., 2019; Hill et al., 2020; Cavanagh et al., 2020).

One notable result from this study is that the *levels* of burnout and secondary traumatic stress observed in dog trainers are broadly similar to those reported in other animal-related and human healthcare professions. Although the focus was not on prevalence per se, it is instructive to contextualize the sample’s ProQOL scores with published data from comparable groups (see Figure 1).

Cavanagh et al. (2020) provide a systematic review and meta-analysis of compassion fatigue among healthcare providers. They reported that mean ProQOL scores typically fell in the moderate range for burnout and secondary traumatic stress and relatively high for compassion satisfaction across various healthcare practitioners. For instance, studies using ProQOL5 found combined average scores of around M ~28 for burnout and ~26 for STS, with compassion satisfaction, with means around 40–42. The dog trainer sample had a mean burnout score of 25.4 and a secondary traumatic stress score of 26.7, with a compassion satisfaction score of 40.1—remarkably close to these healthcare benchmarks. This suggests that dog trainers experience a degree of occupational stress that is on par with nurses, physicians, or social workers in terms of burnout and secondary trauma. At the same time, their compassion satisfaction is also comparably high, meaning they derive significant fulfillment from their jobs, much like many caregiving professionals do as a counterbalance to stress. This parallel is intriguing because one might intuitively expect “caring for humans” to be more emotionally taxing than “caring for animals,” but clearly, the human–animal caregiving context carries its own profound emotional demands.

In the animal-care industry, Scotney et al. (2019) investigated compassion satisfaction, burnout, and secondary traumatic stress among various occupations, including veterinarians, veterinary nurses, shelter workers, and animal research technicians. They similarly found moderate levels of burnout and secondary traumatic stress and relatively high compassion satisfaction across these roles. For example, veterinary personnel often had burnout scores in the 24–27 range and secondary traumatic stress scores around 24–25, with compassion satisfaction scores around 39–40 (Scotney et al., 2019). Those figures mirror what was found in the sample of dog trainers. Scotney et al. found that occupation alone (veterinarian vs. vet nurse vs. shelter worker, etc.) did not significantly predict burnout or secondary traumatic stress risk. Instead, workplace factors and individual differences were pivotal. This resonates with the finding that simply being a “trainer who handles aggression” vs. “trainer who does not” did not dictate burnout; other factors (like work conditions) likely cut across those categories.

Another comparative point: Scotney et al. (2019) observed that longer years of experience in their sample were associated with a greater risk of burnout (and interestingly, being female was associated with higher secondary traumatic stress). The study did not replicate the experience effect for trainers, as discussed, which could be due to the smaller sample or unique aspects of the trainer role. It may also be that in the Scotney et al. study, they had a wider range of organizational contexts (from research labs to clinics) where systemic issues might accumulate over time. Employment context is a critical determinant of how dog trainers and behavior professionals experience occupational demands and psychological outcomes. Self-employed trainers often benefit from greater autonomy over their schedules, client selection, and work methods, which can provide some protection against organizational stressors such as inflexible policies or lack of supervisory support (Hunsaker et al., 2015; Scotney et al., 2019). However, self-employment may also introduce unique pressures, including financial insecurity, the responsibility for all aspects of running a business (e.g., marketing, client acquisition, administrative duties), and potential professional isolation. In contrast, individuals employed by organizations or shelters may have access to established support systems, opportunities for peer collaboration, and structured procedures, but may also face challenges related to limited control over caseloads, rigid institutional protocols, or organizational cultures that do not prioritize employee well-being (Sinclair et al., 2017; Scotney et al., 2019). These distinct experiences highlight the importance of tailoring interventions for compassion fatigue and burnout to the employment context and underscore the need for future research to systematically assess and compare these groups within the animal-care sector.

Looking at other specialized animal-care roles, Jensvold (2022) studied chimpanzee caregivers and found they had even higher burnout and secondary traumatic stress levels (means ~27.5 each) than many other animal professions, despite also high compassion satisfaction. They, like dog trainers, did not show a correlation between experience and burnout. Similarly, Hill et al. (2020) surveyed nearly 2900 animal-care workers (veterinarians, techs, animal control officers) and found moderate burnout (~26.8) and secondary traumatic stress (~25.6) across the board. They noted no significant differences in burnout or secondary traumatic stress between those occupations, underscoring that many caregiving roles (human or animal) converge in the moderate burnout realm. Hill et al. (2020) also found that factors like perceiving the work as a “lifelong dream” were protective and that variables like age or years of experience had variable correlations with burnout/secondary traumatic stress depending on other conditions. Those findings align with the suggestion that a personal sense of mission and job satisfaction (high among trainers) can buffer compassion fatigue. Indeed, one could interpret the trainers’ high compassion satisfaction as a key reason why, despite dealing with trauma and stress, their burnout stayed moderate rather than high.

Further contextualizing these results, Andrukonis et al. (2020) examined animal shelter employees in the United States and found mean ProQOL scores of 37.94 for compassion satisfaction, 27.06 for burnout, and 23.75 for secondary traumatic stress. These values align closely with the current sample of dog trainers, who also demonstrated moderate levels of burnout and secondary traumatic stress alongside high compassion satisfaction. Like the findings for veterinary personnel and shelter workers in Scotney et al. (2019), shelter staff in Andrukonis et al. (2020) experienced emotional rewards from their work (as reflected by relatively high compassion satisfaction) but continued to face notable occupational stressors. The burnout mean for shelter workers (27.06) is just slightly higher than that typically observed in trainers, suggesting both roles navigate persistent workplace challenges—albeit possibly shaped by different daily realities, such as the demands of animal intake and euthanasia in shelters versus client management and behavioral consulting in training.

Moreover, as was the case in Scotney et al., the data from Andrukonis et al. (2020) reinforce the idea that compassion fatigue and burnout are not exclusive to one animal-care sector but represent cross-cutting risks wherever individuals are exposed to emotionally taxing animal welfare responsibilities. The similarity in profiles supports the conclusion that the mental health and well-being of dog trainers are equally deserving of research and intervention as those of shelter staff, veterinary professionals, and other caregiving occupations. It also echoes the notion that interventions that are effective in shelter environments, such as peer support, debriefing protocols, and professional development, may have potential value for the dog training profession. Collectively, these comparative findings emphasize that dog trainers, though historically overlooked, face emotional challenges that are on par with those reported in the broader animal-care sector, and their welfare should be a key consideration in ongoing occupational health research.

In this study, dog trainers exhibited a Professional Quality of Life profile similar to other caregiving professionals, with moderate compassion fatigue, burnout, and high compassion satisfaction. This comparability highlights that the welfare of dog trainers deserves the same attention as that of nurses, therapists, or shelter workers regarding mental health. Historically, dog trainers have been overlooked in research, perhaps seen as outside the traditional “caring professions,” but these data suggest their emotional challenges are not lesser. The universal nature of compassion fatigue and burnout across caring roles means that successful interventions in one domain might also apply to dog trainers (Cavanagh et al., 2020). It also underscores the validity of using instruments like ProQOL in this field—the fact that trainers’ scores align with known benchmarks gives confidence that the instrument captured their experiences meaningfully.

### 4.5. Theoretical and Practical Implications

Although the hypotheses on specific predictors were not supported, the study yields important insights to guide mental health interventions and support strategies for dog trainers. The finding that burnout levels are moderate and compassion satisfaction high suggests that many trainers find their work rewarding despite stress—a strength to build upon. At the same time, the significant role of compassion fatigue indicates a clear target for intervention. Implications for both individual-focused and organizational (or community) interventions, including coaching and support programs, that could help prevent and alleviate burnout in this profession will be discussed below. There will also be an emphasis on the need for a proactive approach to mental well-being among dog trainers, analogous to efforts in other helping fields.

#### 4.5.1. Support and Resilience Training

One key implication is that fostering resilience and healthy coping strategies is critical for dog trainers at all career stages. Stronger support is needed since years of experience alone do not guarantee lower burnout. Training programs or workshops on managing compassion fatigue could be highly beneficial (Rank et al., 2009). For example, teaching trainers about the signs of burnout and secondary trauma and equipping them with coping techniques (e.g., mindfulness, relaxation exercises, cognitive reframing) can empower them to handle the emotional load (Rohlf, 2018). Compassion fatigue prevention training has shown promise in animal-care workers. Rank et al. (2009) describe “training as a treatment” for compassion fatigue in animal caregivers, suggesting that structured educational interventions can reduce compassion fatigue symptoms. These might include seminars on stress management, building self-awareness of emotional states, and peer discussion sessions. The findings that compassion fatigue strongly correlates with burnout imply that an intervention reducing compassion fatigue could also likely lower burnout.

#### 4.5.2. Coaching and Peer Support Interventions

Introducing formal or informal mentoring and peer support programs could mitigate feelings of isolation and provide outlets for stress. For instance, less experienced trainers could be paired with veteran mentors (who are not burned out) to discuss difficult cases and emotional challenges, which serves as both a coaching intervention and relational support. Even though the data did not show that experience reduces burnout, experienced mentors can share effective coping strategies and normalize the struggles that newer trainers face, potentially preventing early-career burnout. Peer support groups—in-person meetups or online communities—can function as a space to debrief tough cases (much like supervision in counseling). Sharing experiences in a safe, non-judgmental setting helps alleviate stress and provides practical advice. In many caregiving fields, peer support interventions have been linked to reduced compassion fatigue and higher job satisfaction (Meadors et al., 2009). Dog trainers could benefit from similar groups, perhaps organized through professional associations (e.g., monthly support calls or forums moderated by a seasoned trainer or a counselor).

#### 4.5.3. Mental Health Resources and Counseling

Given the significant “emotional labor” involved in dog training (managing not just dogs but anxious or grieving owners), trainers would benefit from access to mental health services. Employee Assistance Programs (EAPs) or counseling services are standard in healthcare and social work settings (Joseph et al., 2018); however, many dog trainers are self-employed or in small businesses that lack such programs. Professional organizations and certification bodies in dog training could consider offering resources such as confidential helplines, lists of therapists knowledgeable about compassion fatigue, or partnerships with counselors who provide discounted sessions to trainers. Short-term counseling interventions (for example, cognitive-behavioral therapy focused on stress management) have proven effective in reducing burnout in similar populations (Rohlf, 2018). Encouraging trainers to seek support when overwhelmed—and reducing the stigma about needing help—is crucial.

#### 4.5.4. Work–Life Balance and Self-Care

Interventions should also target the organizational and work practice levels. While work hours or workload were not explicitly studied, it is known anecdotally that many dog trainers juggle irregular hours (clients on evenings/weekends), physically demanding work, and sometimes financial instability. Encouraging a healthy work–life balance is important: trainers should be advised and coached to set boundaries (e.g., not checking client messages late at night, scheduling regular days off, limiting the number of severe cases at one time if possible). Self-care is often emphasized in combating burnout; Lloyd and Campion (2017) discuss its importance for veterinary nurses. For dog trainers, self-care could include taking breaks between clients to decompress, engaging in hobbies unrelated to dogs to recharge, exercise, or mindfulness practices. While these may seem generic, they are frequently neglected in professions driven by passion. Ensuring trainers give themselves permission to rest and recover is something that can be reinforced through coaching. A professional coach or supervisor could work one-on-one with trainers to identify stressors and create personalized self-care plans, acting as a preventive measure. There is emerging evidence that executive or wellness coaching interventions in workplace settings can reduce burnout by improving coping and goal-setting (Hines-Stellisch et al., 2024; Olsen & Nesbitt, 2010). Applying this to dog trainers might involve coaches helping them streamline their business to reduce stress, improve client communication to prevent frustration, and incorporate routines that maintain well-being.

#### 4.5.5. Organizational Culture and Education

For trainers working within organizations (e.g., large training schools, shelters, or veterinary practices that offer training), those organizations should cultivate a culture that acknowledges compassion fatigue and actively works to reduce it (Pavan et al., 2020). This could mean providing regular debriefings after difficult cases, rotating particularly demanding assignments so no one trainer is constantly handling the most challenging cases, and ensuring staffing is sufficient to prevent overload. Leadership should be trained to recognize signs of burnout in their staff and respond supportively (Briggs et al., 2024). The findings that compassion satisfaction is high suggest that trainers value their impact; organizations can further enhance compassion satisfaction by recognizing and celebrating successes (however small) and reminding staff of the positive outcomes they achieve, which can buffer stress (Cavanagh et al., 2020).

#### 4.5.6. Use of Empirically Supported Interventions

It is worth noting that interventions for occupational stress in animal-care professionals, though limited in research, have shown some benefits in preliminary studies. Rohlf (2018) systematically reviewed such programs and found that many focus on psychoeducation and building individual coping resources (e.g., communication skills, relaxation techniques, and self-reflection). These are often delivered in workshop formats. Additionally, borrowing from other sectors with well-studied stress interventions is recommended; cognitive-behavioral therapy (CBT) based interventions and mindfulness-based stress reduction (MBSR) have been effective in healthcare and could be adapted for animal-care workers (Rohlf, 2018). A combination approach that includes education about stress, training in coping skills (CBT techniques to challenge negative thoughts, mindfulness to center oneself after a tough case, etc.), and opportunities for relaxation and reflection (perhaps group yoga or meditation for interested trainers) could be a promising comprehensive intervention. Such interventions should be tested in the dog training community.

Importantly, interventions should not only focus on the individual (“fix the person”) but also consider organizational changes. Le Blanc and Schaufeli (2008) note that a mixture of individual and organizational-level strategies is likely most effective for burnout. Organizational interventions might include ensuring fair compensation (financial strain can exacerbate burnout), reasonable scheduling, providing resources like safe training facilities and equipment for dealing with aggressive dogs (reducing physical danger stress), and fostering a team atmosphere even among independent contractors (perhaps through networks or guilds). For independent trainers, the “organization” might be their professional association or local community—these entities can create structures (like a code of ethics that encourages breaks after euthanasia cases or annual mental health workshops at conferences) collectively supporting trainers.

### 4.6. Limitations and Future Research

Several limitations of this study should be acknowledged when interpreting the results. First, the sample was obtained through convenience and volunteer recruitment, which may introduce self-selection bias. Dog trainers who chose to participate might be those particularly interested in burnout or those experiencing it, or conversely, those who felt comfortable reporting about it. One limitation of the present study is that the demographic questionnaire did not include an explicit response option for the IAABC Certified Dog Behavior Consultant (CDBC) credential. As a result, individuals holding this widely recognized advanced certification may not have been able to accurately report their credential status, leading to their apparent absence in the sample. Future studies should ensure that survey instruments include all major professional certifications, such as IAABC-CDBC, as distinct response options to more comprehensively capture the range of qualifications within the dog training and behavior consultant community. The sample was also restricted to trainers using positive reinforcement, which limits generalizability. Trainers who use other methods might have different experiences of stress. Also, the majority of the participants were female (over 80%), which reflects the gender distribution often seen in the pet training industry, but it meant gender differences could not be examined. Some research suggests female professionals report higher compassion fatigue (Scotney et al., 2019); the study was underpowered to test that, given the imbalance.

Another limitation is the cross-sectional and self-report nature of the data. Causal inferences cannot be made—for instance, while it can be debated that compassion fatigue contributes to burnout, it could also be that feeling burned out makes one more susceptible to compassion fatigue perceptions, or that a third factor (like personal trauma history) increases both. Longitudinal research would help disentangle directions of influence and track how burnout and compassion fatigue evolve over a trainer’s career (for example, do they peak at certain times or fluctuate with workload?). Additionally, all measures were self-reported; while the ProQOL is validated, responses could be influenced by mood or social desirability. Some trainers may underreport burnout due to stigma or overreport compassion satisfaction out of genuine love for their job, skewing the results. Including qualitative interviews could provide depth and identify stressors or coping strategies not captured in numeric scales. The recommendation is that future studies incorporate qualitative approaches; interviews with dog trainers could reveal nuanced stress factors (like difficult client interactions, financial pressures, etc.) and perhaps highlight positive practices used by those who cope well.

The use of the ProQOL’s Secondary Traumatic Stress (STS) subscale as a proxy for compassion fatigue represents a limitation, as it primarily captures the personal, trauma-related aspect of CF and does not fully address the broader, organizational or systemic contributors emphasized in more recent theoretical models (Heritage et al., 2018; Sinclair et al., 2017). Future research should consider employing instruments or approaches that distinguish between individual and organizational components of compassion fatigue to more comprehensively assess this construct.

Another consideration is that the measure of aggression exposure was somewhat coarse (categorical based on self-report percentages). Future research could quantify exposure more granularly and also consider the emotional impact of specific cases rather than just frequency. For example, a particularly traumatic incident (like a severe dog bite to a client) could cause burnout/secondary traumatic stress, irrespective of the overall experience. Including measures of critical incident stress (whether someone has experienced a very traumatic work event) might predict compassion fatigue better than just the number of aggression cases. Also, capturing work context (solo trainer vs. part of a team, urban vs. rural clientele, etc.) could shed light on available support systems.

An important limitation is that the study did not capture or analyze whether trainers were self-employed/operated their own business, or worked as employees within a company, animal shelter, or training organization. Employment status could substantially influence occupational stressors: business owners or self-employed trainers may experience unique pressures related to business management, client acquisition, finances, and administrative responsibilities, in addition to training-related stress. Conversely, trainers working for organizations or shelters may face different forms of stress, such as workload demands, organizational culture, or less control over case selection. Previous research in animal-care and human healthcare professions has noted that management or administrative roles can increase burnout risk, as can a lack of autonomy in employee roles (Cavanagh et al., 2020; Hill et al., 2020). Future studies should collect employment context variables and investigate how organizational role and employment status contribute to burnout and compassion fatigue in dog trainers and behavior consultants. Understanding these distinctions could help tailor interventions to the unique stress profiles of self-employed versus organizationally employed trainers.

Despite these limitations, this study provides a first look at variables that are and are not correlated with burnout and compassion fatigue among dog training professionals. It opens several avenues for future research: (a) exploring other predictors of burnout, such as personality traits (e.g., empathy levels, perfectionism—which is noted as a stress factor in veterinary nurses (Thompson-Hughes, 2019)), social support, client demographics, or business-related stress; (b) implementing and evaluating interventions—for example, testing a resilience training workshop or a peer support program in a randomized controlled trial to see if it lowers ProQOL burnout scores over time (as suggested in the recommendations); (c) conducting longitudinal studies to see how trainers’ burnout trajectories change, especially given events like the COVID-19 pandemic which greatly increased pet ownership and possibly the demand for trainers (but also perhaps stress due to new types of client issues); and (d) expanding to international samples or comparing different training methodologies to see if certain professional subcultures have differing burnout experiences.

## 5. Conclusions

This study is among the first to empirically examine burnout and compassion fatigue in the dog training profession. The study investigated whether burnout in dog trainers is associated with years of experience and the severity of dog behavior cases (aggression) and whether the emotional toll of caring (compassion fatigue) moderates these effects. The quantitative results indicated that neither longer experience nor higher aggression exposure were significant predictors of burnout in this sample, and compassion fatigue did not act as a moderator between those work factors and burnout. In essence, more experience did not equate to more (or less) burnout, and working with aggressive dogs was not by itself a determinant of burnout levels. Compassion fatigue emerged as a strong correlate of burnout overall—trainers experiencing higher secondary traumatic stress showed higher burnout—but this influence was uniform rather than conditional. These findings suggest that factors beyond years on the job and case type (such as personal coping resources, social support, and organizational environment) likely play a crucial role in burnout among dog trainers.

Despite not supporting the initial hypotheses, the study yields valuable insights. Dog trainers exhibited moderate burnout and compassion fatigue levels comparable to those found in other caregiving professions, accompanied by high compassion satisfaction. This indicates that the intrinsic rewards of working with dogs and owners may provide a protective buffer against extreme burnout. At the same time, the emotional challenges inherent in the profession—empathizing with clients’ struggles and animals’ traumas—remain ever-present and must be addressed proactively. The unique combination of challenges dog trainers face (operating at the intersection of human and animal welfare) highlights the need for tailored support and intervention strategies to ensure their well-being. From a practical standpoint, the findings underscore the importance of developing a holistic support system for dog trainers and behavior consultants. This includes fostering peer support networks, providing education on compassion fatigue and self-care, and encouraging the use of mental health resources without stigma. Future research and industry initiatives should focus on creating and evaluating specific interventions—such as resilience training programs, coaching/mentorship arrangements, and organizational policy changes—to reduce burnout and secondary traumatic stress in this field. We can promote a healthier, more sustainable work environment by addressing dog training professionals’ emotional and psychological needs. This benefits the trainers themselves and translates into better outcomes for the dogs and clients they serve, as well as the broader goal of improving human–animal relationships. Ultimately, ensuring the well-being of those “behind the leash” is a critical component of compassionate and effective dog training practice, warranting continued attention and action from researchers and practitioner communities.

## Figures and Tables

**Table 1:** Demographic characteristics of participants (Malone, 2024).

Characteristic	Frequency	%
Gender identity		
Man	6	7.0
Woman	70	81.4
Transgender	3	3.5
Non-binary	5	5.8
Prefer not to answer	1	1.2
Other	1	1.2
Education level		
Some college, no degree	5	5.8
Associate degree	8	9.3
Bachelor’s degree	44	51.2
Master’s degree	24	27.9
Doctorate	1	1.2
Other	1	4.7
Dog training certification		
CPDT-KA	48	56
KPT-CTP	15	17
CTC	10	12
IAABC-ADT	15	17
CBCC-KA	20	23
No certifications	10	12
Training type		
Obedience only	10	11.6
Reactivity only	7	8.1
Both reactivity and obedience	63	73.3
Other	6	7.0
Training method		
R+	82	95
Clicker	44	51
E-collar	0	0
Dominance	0	0
Other	17	20

Notes. Reactivity cases include bite and no-bite cases.

**Table 2:** Cross-tabulation of CBCC-KA certification status and aggression case exposure (bite level).

CBCC-KA	Bite Level 0	%	Bite Level 1	%	Bite Level > 1	%	Total
No (n = 66)	17	25.8	35	53	14	21.2	66
Yes (n = 20)	0	0	15	75	5	25	20
Total (N = 86)	17	19.8	50	58.1	19	22.1	86

**Table 3:** Descriptive statistics for ProQOL scales (Malone, 2024).

Scale	Min	Max	M	SD	Cronbach’s α	Level
Burnout	15	35	25.41	4.983	0.888	Moderate
Comp. satisfaction	29	49	40.12	5.221	0.731	Moderate
Secondary TS	11	41	26.66	6.507	0.839	Moderate

**Table 4:** Summary statistics with Pearson correlation scores (Malone, 2024).

Variables	M	ST	Pearson	*p*
Years of experience	12.98	9.798	−0.080	0.463
Bite level	1.02	0.650	0.055	0.614
Burnout	25.41	4.983		

**Table 5:** Multiple regression analysis results with YE as IV (Malone, 2024).

	Coefficients					95% CI			Collinearity	
	B	SE	β	t	*p*	For B		*sr* ^2^	T	VIF
Constant	13.184	1.068		12.343	<0.001	11.059	15.309			
YE	0.049	0.065	−0.096	−0.759	0.450	−0.178	0.079	0.0021	0.110	9.077
CF	0.005	0.000	0.859	11.413	<0.001	0.709	1.009	0.2313	0.314	3.186
CF × YE	2.910 × 10^−5^	0.000	0.147	1.093	0.278	0.000	0.000	0.0021	0.098	10.199

Notes. YE = years of experience, CF = compassion fatigue, CF × YE = interaction between compassion fatigue and years of experience.

**Table 6:** Multiple regression analysis results with BL as IV (Malone, 2024).

	Coefficients					95% CI			Collinearity	
	B	SE	β	t	*p*	For B		*sr* ^2^	T	VIF
Constant	13.143	1.009		13.025	<0.001	11.135	15.150			
BL	0.716	0.932	−0.093	−0.768	0.445	−2.570	1.139	0.0010	0.117	8.575
CF	0.005	0.000	0.933	13.061	<0.001	0.004	0.006	0.2948	0.338	2.956
CF × BL	2.910 × 10^−5^	0.000	0.147	1.093	0.278	0.000	0.000	0.0021	0.098	10.199

Notes. BL = bite level, CF = compassion fatigue, CF × BL = interaction between compassion fatigue and bite level.

**Table 7:** Multiple regression analysis results with YE and BL as IV (Malone, 2024).

	Coefficients					95% CI			Collinearity	
	B	SE	β	t	*p*	For B		*sr* ^2^	T	VIF
Constant	13.609	1.012		13.451	<0.001	11.596	15.662			
BL	−1.044	0.439	−0.136	−2.380	0.020	−1.917	−0.171	0.0096	0.518	1.930
YE	−0.010	0.033	−0.020	−0.304	0.762	−0.077	0.056	0.0001	0.392	2.549
CF	0.005	0.000	0.909	18.160	<0.001	0.004	0.005	0.5595	0.678	1.476
CF × BL × YE	1.053 × 10^−5^	0.000	0.097	1.179	0.242	0.000	0.000	0.0024	0.253	3.957

Notes. BL = bite level, CF = compassion fatigue, YE = years of experience, CF × BL × YE = interaction between compassion fatigue, bite level, and years of experience.

**Table 8:** Summary of hypotheses and results.

Hypothesis No.	Hypothesis Description	Supported by Data?
H1	Burnout will vary with years of experience on the job among trainers and behavior consultants who offer obedience and aggression training for dogs.	No
H2	Burnout will vary among trainers and behavior consultants who offer obedience and aggression training for dogs in relation to the aggression levels of target dogs.	No
H3	Compassion fatigue moderates the relationship between years of experience and burnout.	No
H4	Compassion fatigue moderates the relationship between the aggression level of dogs and burnout.	No
H5	Compassion fatigue moderates the relationship between years of experience, aggression level, and burnout.	No

## Data Availability

The data presented in this study are available on request from the corresponding author due to privacy restrictions expected by participants when they agreed to participate in the study.
